# Collective Reflective Equilibrium in Practice (CREP) and controversial novel technologies

**DOI:** 10.1111/bioe.12869

**Published:** 2021-05-04

**Authors:** Julian Savulescu, Christopher Gyngell, Guy Kahane

**Affiliations:** ^1^ Oxford Uehiro Centre for Practical Ethics University of Oxford Oxford United Kingdom of Great Britain and Northern Ireland; ^2^ Wellcome Centre for Ethics and Humanities University of Oxford Oxford United Kingdom of Great Britain and Northern Ireland; ^3^ Biomedical Ethics Research Group Murdoch Children’s Research Institute Parkville Australia; ^4^ Melbourne Law School University of Melbourne Melbourne Australia; ^5^ Department of Paediatrics University of Melbourne Melbourne Australia

**Keywords:** algorithm, artificial intelligence, bias, driverless cars, egalitarianism, ethical decision procedures, policy, reflective equilibrium, utilitarianism, veil of ignorance

## Abstract

In this paper, we investigate how data about public preferences may be used to inform policy around the use of controversial novel technologies, using public preferences about autonomous vehicles (AVs) as a case study. We first summarize the recent ‘Moral Machine’ study, which generated preference data from millions of people regarding how they think AVs should respond to emergency situations. We argue that while such preferences cannot be used to directly inform policy, they should not be disregarded. We defend an approach that we call ‘Collective Reflective Equilibrium in Practice’ (CREP). In CREP, data on public attitudes function as an input into a deliberative process that looks for coherence between attitudes, behaviours and competing ethical principles. We argue that in cases of reasonable moral disagreement, data on public attitudes should play a much greater role in shaping policies than in areas of ethical consensus. We apply CREP to some of the global preferences about AVs uncovered by the Moral Machines study. We intend this discussion both as a substantive contribution to the debate about the programming of ethical AVs, and as an illustration of how CREP works. We argue that CREP provides a principled way of using some public preferences as an input for policy, while justifiably disregarding others.

## 1 INTRODUCTION

Radical technological advances such as artificial intelligence and genome editing present profound ethical and policy challenges. There is currently widespread disagreement about how to regulate these technologies, as well as about the fundamental ethical principles that should guide regulation. There are no clear relevant precedents we can apply; nor can we rely on the lessons of long experience. Making policies to oversee novel technologies, in the face of such political and ethical disagreement, is therefore a preeminent challenge facing contemporary society.

One development that suggests a possible solution is our growing capacity to collect data about the public’s attitudes toward novel technologies and the policies that might regulate them. While surveys about public attitudes to new technologies and ethical issues have a long history, it is now possible to use the internet to quickly gather information from millions of people around the world, generating highly robust data about global views about different policy options. We are thus entering an age in which we will have access to unprecedented amounts of data on public attitudes and preferences. How should this information inform policy‐making for novel technologies?

One area where this issue is imminently important is the programming of autonomous vehicles. Autonomous vehicles (AVs) will confront situations in which bad outcomes are inevitable. For example, if a pedestrian walks in the path of the car, the car may have to either swerve into traffic and risk the life of the driver, or continue and risk the life of the pedestrian. The need to decide how AVs should respond to such situations is stimulating research into machine ethics.

In this paper, we will approach the general issue of how data about public preferences may be used to inform ethical policy by looking closely at the specific question of how public preferences about AVs ought to inform their programming. In Section 2 we summarize the recent ‘Moral Machines study’, which collected data from millions of people on how they think AVs should respond to emergency situations. In Section 3, we turn to ask how this data should be used. We consider two extreme views: (1) AVs should be programmed to completely align with public attitudes; (2) public attitudes should have no bearing on policy. We will argue that neither view is plausible. In Section 4, we defend an alternative approach that we call ‘Collective Reflective Equilibrium in Practice’ (CREP).^1^ In CREP, data on public attitudes can have an important role to play in shaping policy but only as potential input into a deliberative process that looks for coherence between attitudes, behaviours, and ethical principles. We argue that in cases of reasonable moral disagreement, data on public attitudes should play a much greater role in shaping policy than in areas of ethical consensus. In Section 5, we apply CREP to some of the global preferences about AVs uncovered by the Moral Machines study. We intend this discussion both as a substantive contribution to the debate about the programming of ethical AVs, and as an illustration of how CREP works in relation to novel technologies. We argue that CREP provides a principled way of using some public preferences as an input for forming concrete policy that is practically implementable, politically legitimate, and ethically defensible, while justifiably disregarding other public preferences.

## 2 AUTONOMOUS VEHICLES AND THE MORAL MACHINES STUDY

Several major car manufacturers have announced plans to release fully driverless cars (with no steering wheel or gas pedal) by 2024.^2^ These AVs are expected to produce many benefits.^3^ For individuals, AVs promise increased mobility, reduced stress, and increased safety. More broadly, AVs promise safer roads, less pollution, and reduced congestion.

However, AVs also present challenges to policy.^4^ One such challenge is how to program AVs to respond in emergency situations where death is imminent. This challenge is notable for turning an abstract philosophical debate that has been ongoing for over five decades into a concrete ethical conundrum.

In 1967, Phillipa Foot introduced the first ‘Trolley’ dilemma.^5^ In this scenario, a trolley will kill one group of people if no action is taken, but it is possible to divert the vehicle so that it would instead hit a second group. Asking when it is, and is not, permissible to divert the trolley is an effective way to probe intuitions about the moral permissibility of sacrificing one group of people in order to spare another. Trolley dilemmas are very flexible, as the groups involved can contain any number of people, different types of people (pregnant women, doctors, homeless, criminals, etc.), and even animals.

It is a common criticism of such thought experiments involving trolleys that they describe far‐fetched scenarios that are claimed to bear little resemblance to any real‐life ethical decision.^6^ However, AVs *will* confront situations that closely resemble trolley dilemmas. For example, if a woman pushing a baby in a stroller walks into the path of the car, AVs may be forced to make a choice between continuing straight, risking the life of mother and child, or to swerve, risking the life of a single pedestrian or the occupant/s of the vehicle, if there is insufficient time to brake effectively. AV programmers will not directly confront trolley dilemmas—as they will not be literally pulling a lever to divert the AV, and they can only shape the ‘decisions’ of the AV indirectly, via their prior programming.^7^ However, AV programmers will need to assign value to possible classes of entities that an AV may encounter in contexts where split‐second decisions must be made about the distribution of risk and harm. In a sense, AVs must be programmed with a sort of ‘moral status detector’, which allows them to differentially respond to a backpack, an empty car, a dog, and a human—as well as, perhaps, even to different classes of humans (old vs. young, pregnant vs. non‐pregnant, etc.; see below)

### Public attitudes toward AVs and trolley problems

In one of the largest studies ever performed on global moral preferences, Awad et al. collected data on people’s preferences to various trolley‐like dilemmas involving AVs, generating over 40 million data points.^8^ They identified nine factors that typically led participants to spare one group over another. These were, in decreasing order of strength, a preference for:

- Sparing human lives over animal lives
- Sparing more lives rather than fewer lives
- Sparing the young over the elderly
- Sparing the law‐abiding over law‐breakers
- Sparing those of high social status over those of low social status
- Sparing those who are a healthy weight over those who are overweight
- Sparing females over males
- Sparing pedestrians over passengers
- Preferring the vehicle to continue in its motion, over it taking evasive action.

People from all over the world shared these preferences. The study also identified three cultural clusters that gave different weights to different preferences. These were a Western cluster (broadly North America, Europe and Commonwealth countries), an Eastern cluster (broadly Asian countries), and a Southern cluster (broadly Central and South America, and territories that were at some point under French leadership).

Participants from the Western cluster were much more likely to show a strong preference for saving the many over the few than those in other clusters. The preference to spare the young rather than the old was much less pronounced for countries in the Eastern cluster, and much higher for countries in the Southern cluster. Finally, those from the Southern cluster showed a much stronger preference for saving females and those who were physically fit. These preferences were highly correlated with cultural and economic differences between countries.

## 3 SHOULD PUBLIC PREFERENCES INFORM POLICY?

Awad et al. conclude their paper by writing that

Our data helped us to identify three strong preferences that can serve as building blocks for discussions of universal machine ethics, even if they are not ultimately endorsed by policymakers: the preference for sparing human lives, the preference for sparing more lives, and the preference for sparing young lives.^9^

Our aim in what follows is to clarify whether, and in what way, such global preferences can serve as building blocks for policy.

### 3.1 Should policymakers endorse the preferences uncovered by the Moral Machine experiment?

We can start by asking which of the nine preferences identified by the experiment should inform the programming of driverless cars. As the quote above indicates, Awad et al. appear to believe that only the three *strongest* preferences should be used. However, there are several problems with this.^10^

For one, the three preferences Awad et al. identify (humans over animals, saving the greatest number, and saving the young) are not equally strong in all regions. For example, in Eastern regions, the preference for young lives over older lives was weaker than the preference for saving the lawful. In Southern regions, sparing individuals of high social status was nearly as strong as the preference for saving the young. If we make decisions about which preferences to program into AVs based on which are strongest, we will need to decide whether we should choose the strongest preferences overall, or the just the strongest in the particular jurisdiction where the AV will operate.

More fundamentally, it is clear that some preferences should not inform policy no matter how strong they are. Participants in the Awad et al. study were not asked whether they would prefer to save members of their own ethnic group over those from different ethnicities, or if they would prefer compatriots over tourists, or members of their own religious group over other religious groups. The public is very likely to have such preferences given that smaller‐scale studies employing trolley dilemmas have found that many people do prioritize compatriots over foreigners, relatives over strangers, and in some cases even discriminate on the basis of race, social class or disability.^11^ Yet it goes without saying that a preference for saving one’s own ethnic group should be ignored in moral and policy decision‐making, no matter how strong it is.

This is merely one instance of a general worry about relying on public preferences to shape policy. Very many views that we now consider paradigmatic forms of prejudice or deep error—for example views about the subordinate role of females, the superiority of ‘white’ people, or about how ‘heretics’ should be treated—used to be widely, and in some cases almost universally, held until not long ago. It might be argued that whatever moral progress we have made so far—including the emancipation of women, the abolishment of the slave trade, increasing concern for animal welfare—is due precisely to using reason, and careful ethical theorizing, to challenge common intuitions. Future people may well come to view some of our present intuitions—for example about prioritizing humans over animals—as similarly prejudiced. As Peter Singer forcefully writes,

all the particular moral judgments we intuitively make are likely to derive from discarded religious systems, from warped views of sex and bodily functions, or from customs necessary for the survival of the group in social and economic circumstances that now lie in the distant past…^12^

Moreover, even when our moral intuitions do not have such problematic sources, extensive psychological research has been taken to suggest that our moral intuitions are often highly unreliable.^13^ Research has shown, for example, that people’s responses to trolley dilemmas may be influenced by morally irrelevant factors such as their current mood^14^ or framing effects.^15^ Indeed, the influence of such framing effects has even been claimed to be a reason to doubt the significance of strong public support for euthanasia.^16^

A final worry is the familiar one that when policy decisions are made in accordance with majority rule, this enables the exploitation of minorities. Such forms of ‘democratic ethics’ could quickly lead to legal discrimination based on race, religion, and gender orientation—enabling what Mill called ‘the tyranny of the majority’. Mill argued that

there needs protection also against the tyranny of the prevailing opinion and feeling; against the tendency of society to impose, by other means than civil penalties, its own ideas and practices as rules of conduct on those who dissent from them.^17^

To make ethical decisions a matter of referendum is to eschew ethical expertise and professional responsibility. After all, we should expect policy‐makers or bodies to be better informed and more competent moral judges than ordinary folk.

### 3.2 But we cannot ignore public preferences…

If our moral preferences are unreliable and often reflect ingrained biases, perhaps they should have no influence on policy. We could follow Singer’s advice and conclude that ‘it would be best to forget all about our particular moral judgments’.^18^ On this view, the preferences revealed by the Moral Machines study should have no bearing at all on how AVs are programmed. Instead, we should look to moral theory as a guide. This is the line taken by John Harris, who describes the work in the Moral Machine experiment as ‘useless’.^19^ Harris argues that

The idea that it might be open to individual citizens or corporations to decide who shall be “spared” and who condemned to death, and that this might be a matter of mere individual “preference”, made on the basis of the sorts of sampling described in [the Moral Machines paper]… is outrageous in the extreme.^20^

This suggestion, however, also faces serious challenges. First, there is no universally accepted ethical theory that we could simply program AVs to accord to. Although many moral theories embrace certain fundamental moral ideas (such as the equal standing of people), they interpret these ideas in radically different ways. Hence in utilitarianism, equal standing is reflected through counting each person’s happiness and pain equally; in Kantian ethics, equal standing is understood to relate to our common dignity; on contractualist views, equal standing may relate to equality of position behind the veil of ignorance; and egalitarians call for equal exposure to risk.

Different moral theories famously give conflicting answers to many trolley dilemmas. For example, in the classic trolley dilemma, utilitarians hold that we are required to divert a trolley that will kill five to a sidetrack where it will kill only one, as this will achieve the greatest good. Yet some deontologists argue that it is wrong to divert the trolley.^21^ And strict egalitarians should presumably flip a coin to decide if they flip the switch, as that will give each of the six people an equal chance of survival (50%). Since these central theories—as well as individual ethicists—often disagree on fundamental moral questions, it is unclear which of these competing theories should guide policy. Moreover, policymakers might simply appeal to those ethical theories, or supposed ethical experts, that match their preconceived ideas or biases.

In addition, many (and arguably all) ethical theories themselves rely, directly or indirectly, on some moral intuitions, including some intuitions about particular cases.^22^ That is to say, they rely on the intuitions of a handful of moral philosophers. While philosophers may have more refined intuitions than ordinary folk, their background, psychology and life experience may be highly idiosyncratic. And the evidence suggests that philosophers’ intuitions too are susceptible to framing effects.^23^ So we risk replacing the tyranny of the majority with the tyranny of the unusual few. If moral intuitions already play a role in moral decision‐making, then it is hard to see why the intuitions of the general public should be excluded from playing any such role, unless they can be shown to be uniquely untrustworthy.

We are inclined to say something even stronger. With many others, we will assume that moral intuitions, including intuitions about cases (such as those expressed by the preferences collected by Awad et al.), *can* confer justification on moral views and, by extension, on policies that implement those views—*if* these intuitions meet certain conditions that we will discuss below. While this assumption is rejected by Singer and others, it is widely accepted within moral philosophy.^24^ Importantly, such justification is defeasible, and can be lost if the source of an intuition is exposed as prejudice or bias.^25^ One factor, however, that is often taken to give further support to an intuition (if it has not been shown to be biased) is if it is very widely held, even by members of different cultures.^26^ Large‐scale studies such as the Moral Machine offer unprecedented ways of measuring such global convergence.^27^ The question then is how to integrate such evidence about widespread intuitions into ethical reflection, and especially into decisions about the regulation of novel technologies.

There is a further reason why we cannot simply ignore public preferences and intuitions when it comes to making AV policy. In liberal democracies, the legitimacy of a policy depends on whether the public have a role in shaping it. Laws and policies that do not have public support are still legitimate in a democracy, if the public are part of the process through which these laws are created (for example through electing representatives who make the laws). However, if we are discussing which policies to make, then the fact that certain policies do align with public preferences seems to be at least a pro tanto reason in their favour.^28^ This is especially true in the context of emerging technologies. As the World Economic Forum’s 2015 Global Risks report points out, ‘the general public must … be included in an open dialogue about the risks and opportunities of emerging technologies’.^29^ This is also in line with calls for ‘broad societal consensus’^30^ around genome editing.

### 3.3 Free choice

Given the above problems, one might think that a natural solution is to let each individual program their own driverless car. However, there are very good reasons to avoid such an approach. Previous data from the Moral Machines group demonstrate this. In their previous published work on 2000 responses, they found that ‘[s]eventy‐six percent of participants said they would prefer vehicles to respond to impending crashes in a ‘utilitarian’ manner, and choose the action that would save the most lives’.^31^ But ironically, respondents also said that they would ultimately buy a car programmed to preserve their lives as passengers over a utilitarian vehicle. On a willingness‐to‐buy scale of one to 100, subjects rated self‐preservation of themselves and family as a 50, while the decision for self‐sacrifice had a median ranking of 19. Participants also indicated they would be less likely to buy a self‐driving car if the government mandated utilitarian technology.

One can imagine that if people could program their own cars to be sensitive to different demographic features, they would simply program them to save people like themselves, or even more likely, their own family. This would simply be another way of enabling the tyranny of the majority. It would also lead to a collective action problem, where what is in the self‐interest of one leads to what is worst for all.

We need government leadership and laws if we are to solve global collective action problems, such as reducing carbon emissions, but also if we are to introduce driverless cars. Humans have a tendency to free ride on the sacrifices of others. We should not let the market decide. If the public is less likely to buy a more ethical driverless car, they can be incentivized or even coerced. Laws and policies are required to prevent the tragedy of the commons and to ensure that risk of harm is minimized to reasonable levels.

## 4 *A PROPOSAL:* ‘COLLECTIVE REFLECTIVE EQUILIBRIUM IN PRACTICE’

We cannot simply base our policy on the strongest public intuitions; nor can we simply ignore public intuitions in favour of top‐down policy‐making. The challenge, then, is to find a way of integrating ethical theory and data about the spread of public intuitions while minimizing the risks associated with each. In the rest of this paper, we will sketch an ethical decision procedure that we believe can meet this challenge.

In 1951, John Rawls famously outlined a ‘decision procedure for ethics’, in which he proposed a rational method for settling conflicts between competing preferences.^32^ With further refinement, this idea has developed into a widely employed approach to moral justification. On this approach, we should aim to achieve coherence between our considered moral judgments about particular cases, our moral principles and values, and (when the equilibrium is ‘wide’) relevant background theories.^33^ When we approach such reflective equilibrium, our moral views are justified because they enjoy the ‘mutual support of many considerations’.^34^ Although it has faced its share of critics,^35^ the method of reflective equilibrium is widely used across ethics—Thomas Scanlon has even described it as ‘the only defensible method’ because ‘apparent alternatives to it are illusory’.^36^

Although in early work Rawls occasionally described reflective equilibrium as a way for us to collectively arrive at reasonable moral beliefs, this approach is primarily understood as a way for an individual agent to arrive at moral justification.^37^ However, as Arras and Brody point out, ‘in most settings where practical ethics is called for, our reasoning is ideally social rather than individual; we must try to reach some form of working consensus among many parties with a stake in the outcome.’^38^ Yet if individuals have different starting points, it is unlikely that reflective equilibrium will yield any kind of consensus.^39^

**FIGURE 1:**
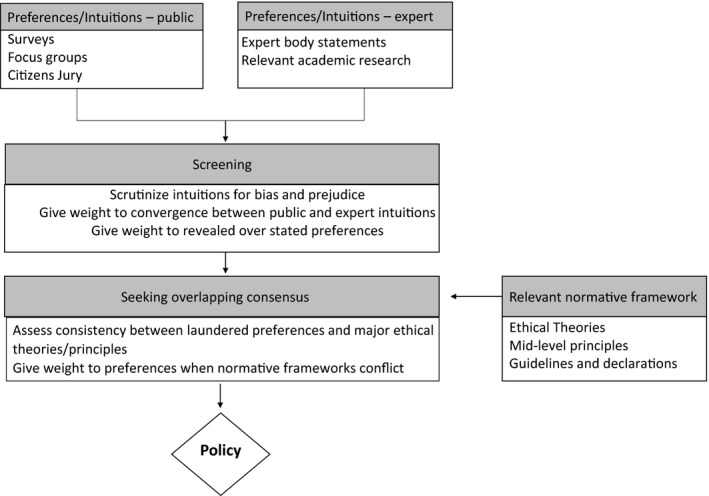
Flowchart of collective reflective equilibrium in practice

The decision procedure that we will propose draws on some key aspects of reflective equilibrium, but instead of focusing on the judgments of a single individual, it uses data about public (and potentially, even global) moral intuitions as input to a process of forming policy to address contentious issues, especially those arising from novel technologies. The aim is to arrive at policy that is both ethically defensible and politically legitimate.^40^ We call this process ‘Collective Reflective Equilibrium in Practice’ (CREP). An overview of CREP can be seen in Figure 1. The key differences between CREP and Rawlsian reflective equilibrium are summarized in Table 1.

**TABLE 1:** Key differences between collective reflective equilibrium in practice and Rawlsian reflective equilibrium as typically practised

	RE	CREP
Deliberators	Abstract category of competent judges; in practice, typically professional ethicists	Policymakers, ethicists, lawyers, relevant medical and scientific experts, lay public and citizens
Intuitional input	Of an individual competent judge	Preferences/judgments of general public/citizen juries
Theoretical input	Ethical theories/principles, primarily those already endorsed by the deliberator (plus relevant background scientific and philosophical theories in wide RE)	Ethical theories, values, principles, frameworks, guidelines, declarations (special weight to either widely shared principles or most ethically justified theories)
Output	Ethical justification for judgments about specific cases and revision of general principles and theories	Justified policy for democratic process
Iteration	Repeated	Limited

Say we need to make policy for a novel technology, and have access to extensive data on public views about different ways that technology might be used. How should we proceed?

As Rawls already emphasized—and as psychological evidence about irrelevant influences confirms—we must not accord weight to just any passing intuition or preference. For Rawls, the starting point was not immediate gut reactions but the considered judgments of competent judges who are impartial, know the relevant facts, possess sympathetic knowledge of the relevant human interests, and who make a serious effort to overcome the sway of prejudice and bias.^41^

Since the procedure we outline takes as its input data about public intuitions, these criteria obviously cannot be directly applied: few ordinary people answering a survey would approximate Rawls’s ideal competent judges, and we cannot guarantee that the preferences we collect always reflect considered, informed judgments. What we can do, however, is to *screen* the initial public preferences to ensure that they are as robust as possible. This means excluding data that we have reason to think are unreliable or not genuinely representative. Here we can draw on our growing knowledge of problematic psychological influences on intuitions. For example, intuitions that are the product of framing effects, or are contingent on a person’s transient mood, will not produce reliable data.^42^ We cannot entirely exclude the effects of such biases but we can ensure, for example, that the data we collect about public preferences for AVs are not subject to order effects and that they are robust to irrelevant changes in the wording or presentation. We must similarly ensure that participants fully understand the relevant factors, and that they are encouraged, and given ample time, to reflect. Finally, we must ensure that the data reflect the views of the population as a whole rather than of some arbitrary or privileged subset.^43^

Thus, in CREP, competent judges are not using their own intuitions—they are applying the reflective equilibrium to the ‘will or preferences of the people’. It is their responsibility to apply ethical theory, principles, concepts and justification to the preferences of people affected by their policy.

Indeed, some survey data, while reliable and representative, may only indicate people’s *stated* preferences, but not their *revealed* preferences. One example of this is survey data from pregnant women, which indicate that less than 45% would terminate a pregnancy if they received a diagnosis of Down syndrome.^44^ However, we know that, in fact, over 90% of women do terminate their pregnancies following a diagnosis of Down syndrome.^45^ Survey data that clearly conflict with people’s actual behaviour offer only a limited basis for policy. In many cases—and especially when novel technology is at issue—we cannot directly gather data about people’s actual behaviour as opposed to their stated views; and gathering such data on a large scale will often be difficult and expensive. However, it may be possible to first investigate, on a smaller scale, the degree to which stated preferences reflect revealed ones. For example, a number of studies have sought to clarify the extent to which responses to abstract trolley dilemmas indeed reflect how people would actually behave in similar situations. Other examples are studies involving realistic decisions about sacrificing animals or using virtual reality that have suggested that people are more willing to sacrifice some to save a greater number than is indicated by responses to hypothetical dilemmas.^46^ Using such means, we could then ‘correct’ larger‐scale data about stated preferences.^47^ Moreover, big data and machine learning offer new prospects for learning what people’s values and preferences are from other related behaviour. For example, ‘a value of life’ could be imputed from the treatment‐limitation behaviour of doctors or from money spent in averting road traffic accidents.

Once we have a set of ‘robust data’ of public attitudes that have been ‘laundered’ in this preliminary way to minimize bias and increase reliability, and to reflect as far as possible true preferences, the second step of CREP is to look for coherence between these intuitions and moral principles. That is, we need to check if these intuitions are actually responding to ethically plausible underlying *reasons,* which would increase our confidence in their validity.^48^ After all, even the most considered and widespread judgments can still be mistaken.

So we need to put these ‘laundered’ intuitions through the deliberative process, a process in which open‐minded, conscientious deliberators—who could but need not be professional ethicists—who meet the Rawlsian criteria we sketched earlier pit these judgements against more general ethical values, principles, and theories. In the context of an individual’s deliberation, the most relevant values, principles and theories are obviously those of the given individual. But even here, Rawls emphasized that we should seek not just internal coherence with our own principles but also thoroughly consider as many reasonable views and arguments that bear on this question as we can. In the context of collective deliberation (or deliberation *for* a collective), there are broadly two ways to identify the relevant theories and principles. If we focus on political legitimacy, we should draw on the principles that are widely shared within (or implicit in the thinking of) the population, perhaps weighted to reflect their popularity. The second approach, which we will favour here, emphasizes moral justification and focuses more on those ethical theories that, after decades of critical reflection, are seen as serious candidates within moral philosophy. But ideally a balance must be struck: giving great weight to theories that bear no relation whatsoever to actual public views will reduce legitimacy, whereas giving weight only to popularity would again open the door to views that are pernicious even if widely held.

Here we must mark an important difference between CREP and traditional reflective equilibrium. Whereas reflective equilibrium involves the repeated adjustments of both particular judgments and general principles, ideally leading an individual both to justified judgments about specific cases and to the singling out of a single justified set of general principles, CREP does not aim to resolve general theoretical disagreement but to identify a legitimate and ethically justified policy. Thus, instead of adjusting our theories to fit our particular judgments across a wide range of contexts, we consider the extent to which common judgments in a specific context—that relating, for example, to the regulation of a novel technology—cohere with the different competing theoretical frameworks and relevant normative guidelines. The idea is that the more a given public preference coheres with more theories and principles, the greater the justification and legitimacy it will have. Why it should have greater legitimacy should be clear: a policy that can be justified via multiple frameworks enjoys what Rawls called ‘overlapping consensus’ and can be endorsed by, and justified to, a greater number of people. With respect to justification, just as so‐called ‘mid‐level’ moral principles have more support if they can be justified by multiple general frameworks, more specific policies gain more support the more they cohere with multiple general frameworks *and* such mid‐level principles. More controversially, adopting the policy that best coheres with most ethical frameworks confers higher‐order justification in the face of moral uncertainty.^49^

According to CREP, we should dismiss policies that neither reflect general intuitions nor cohere with most reasonable ethical frameworks. We should also dismiss intuitions that are rejected by all (or even most) reasonable frameworks even if widely accepted (it will often be obvious that certain intuitions fail this test, and these would already be excluded in the ‘laundering’ stage). We do not, however, have a ready recipe to offer for addressing those cases where a fairly widespread intuition is supported by some theories yet rejected by others; a corresponding policy may have legitimacy but only qualified moral justification. When competing theories conflict, we can also weight them by the degree to which they reflect (or capture) the views of the public.

Relating intuitions about cases and more general theories is rarely a mechanical process. The intuitions need to be clarified, and principles cannot be applied to complex real‐life situations without interpretation. So getting from intuitions and theories to policy recommendations requires deliberation.

The last few decades have seen the development of alternative approaches to democracy, which emphasize increased citizen involvement in policy‐making.^50^ Advocates of participatory democracy aim to achieve breadth of citizen engagement, by including as many people as possible in the policy‐making process, while advocates of deliberative democracy aim to achieve depth in engagement by providing opportunities for small (but representative) groups to engage in meaningful, rigorous and deep deliberation on policy matters.^51^^,^ 52 CREP incorporates elements of both participatory and deliberative approaches. It facilitates participation by a large number of people in policy‐making through the use of large‐scale surveys, and CREP can also incorporate more in‐depth deliberation involving smaller groups of citizens. The latter can help to clarify the content of public intuitions as well as identify more general principles that shape public moral thinking.

In CREP, deliberation occurs in two stages. First, as just mentioned, among members of the public in tools such as citizen’s juries and assemblies, when this is feasible. This then gets fed into the deliberation of ethically informed policymakers. One feature of CREP is that public input is upstream of expert deliberation, which is constrained both by evidence about public intuitions and by prior public deliberation.^53^ Policy makers are not seeking coherence between their *own* intuitions and their favoured ethical principles, but acting as expert representatives of and for the public, seeking coherence between *our* intuitions (potentially weighted for degree of acceptance) and *our* (often conflicting) moral principles. For example, early public engagement activities may identify value conflicts, which have to be considered and analysed in subsequent deliberation by policymakers.

While it is a relatively small group who ultimately make policy decisions in CREP, this is unavoidable in the context of designing policy for novel technologies. For policy matters that are technically complex, involve competing public values, and have wide social implications, in‐depth deliberation is essential to adequately analyse the issues raised.^54^ Furthermore, often specialized knowledge is required to adequately apply the results of ethical analysis in specific contexts. This favours more detailed deliberation between smaller numbers of people, with specific expertise.^55^ This may include expertise in ethics, law, psychology, relevant medicine or science, business, those most affected by the decisions, marginalized groups, ordinary members of the lay public, etc.

We will not discuss further design elements of individual deliberative activities, noting how these issues have been explored in depth elsewhere.^56^ The contribution of CREP is to clarify the role in this process of data about public preferences, on the one hand, and the constraints imposed by ethical theories on the other. Public preferences do not directly decide policy, but serve as a key input in a deliberative process that tests these attitudes against the best established current ethical theories. The judgments that end up shaping policy are not simply those of the majority, but those public attitudes that are widely held, that have been rigorously screened for bias, *and* that are supported by strong moral reasons from converging ethical theories.

## 5 AN APPLICATION: PUBLIC INTUITIONS ABOUT DRIVERLESS CARS

We can now illustrate this approach by applying it to the global preferences about driverless cars collected by Awad et al. To simplify, we will only consider the observed preferences to save the greatest number, to spare the young over the old, and to save females over males. While Awad et al. did not consider disability, this is often a contentious issue in discussions of debates about prioritization and the value of life.^57^ In research one of us has done into public preferences about who should be saved in an emergency context, it was found that people always prioritized the ‘abled’ over the disabled, even when the disability was described as mild.^58^ For illustration, we will also consider this further preference.

Firstly, we should examine the data produced by Awad et al., to see if it robust—that is, reliable and representative. Awad et al. conducted an online survey, with self‐selected participants. This leads to serious concerns about the data being representative, as such a study design is likely to attract certain kinds of participants. Indeed, the authors note that the dataset is skewed towards males in their 20s and 30s. This raises the prospect that the nine preferences identified merely reflect the preferences of young men, rather than being universal. However, the massive size of the dataset helps to alleviate these concerns. Just over 490,000 participants completed the optional demographic survey on age, education, gender, income, and political and religious views. While most responses were from young, educated men, Awad et al. still received many thousands of responses from other groups. This allowed the authors to investigate the role of demographics in the pattern of preferences they observed. They found that ‘individual variations have no sizable impact on any of the nine attributes’. The only statistically significant influences on preferences that was driven by demographic differences were females having a 0.06% stronger preference for sparing females over males, and *religiosity* being associated with a 0.09% higher inclination to spare humans over animals (per standard deviation increase). Importantly, none of the six demographic factors (age, education, gender, income, and political and religious views) were associated with a change in the direction of the nine preferences. We can therefore be reasonably confident that the nine preferences revealed by the study reflect genuine global preferences.

It is possible that the data are affected by framing effects, but again this is somewhat mitigated by the study design. The authors relied on both written and pictorial descriptions of the ethical dilemmas. Some used just written descriptions, and some used just pictorial, yet no difference was found between groups who relied on one rather than the other. Again this suggests that the data reflect people’s robust intuitions about these cases—something close enough to their considered judgments—rather than a transient response to some irrelevant features of the images or wording.

A further question is whether the responses reflect the participants’ actual preferences, rather than just their stated preferences. For some of the intuitions, this seems well supported. Most people behave as if they value saving more lives rather than fewer lives, and in some contexts age is taken to be a relevant factor in saving lives, for example when prioritizing organ transplants. Standard health economic frameworks such as *Quality Adjusted Life Years* (QALYs) similarly give priority to younger people.

In sum, there is reason to think that the data collected by the Moral Machine experiment is likely to accurately represent robust global patterns in public views about the programming of AVs, and that they do not merely reflect framing effects—though, as we shall see below, they may nevertheless reflect other biases.

The next step is to gather relevant ethical theories, concepts and principles, as well as professional guidelines, and to look for consensus and overlap between candidate normative frameworks and the revealed public intuitions. We do not have space to cover all relevant ethical frameworks. But we will briefly consider three of the main ethical approaches: Kantianism, contractualism, and utilitarianism.

The *Kantian* approach is roughly that human dignity requires us to treat all humans equally, not subjecting them to trade‐offs where the rights of some are sacrificed for the good of others. The view that driverless cars should be programmed according to such Kantian principles is reflected in the German Federal Ministry of Transport and Digital Infrastructure’s Ethics Commission.^59^ The German Federal Ministry’s report states: *‘In the event of unavoidable accident situations, any distinction based on personal features (age, gender, physical, or mental constitution) is strictly prohibited.’* This strict egalitarian approach means that *no* personal characteristics (age, sex, or disability) could be used when considering collisions between driverless cars and pedestrians.

On one way of interpreting this view, we should give all involved parties an equal chance of being saved. This, however, is not the approach of the German Ministry report, which instead interprets the view as forbidding AVs from changing course to sacrifice non‐involved parties.^60^ This is in line with the public preference for ‘doing nothing’, though that preference might also merely reflect a status quo bias.

By contrast, *utilitarianism* defines the right act as the act that maximizes utility. As Bentham famously described it, the greatest good for the greatest number. Utilitarians would save the lives that are likely to lead to more utility (well‐being). They would therefore save the greater number, and prioritize younger people (who have more utility in their future) over older people. To the extent that sex does not robustly link to difference in utility, utilitarianism would reject sex as a relevant consideration. Finally, utilitarians often controversially give greater priority to non‐disabled over disabled lives, as does health economics in the form of QALYs.^61^ However, whether this is justified on utilitarian grounds will depend on the relationship between disability and well‐being. While the general public, and many medical practitioners, regard disability as a significant disadvantage, others argue that this assumption reflects ignorance and, indeed, an ‘ableist’ prejudice akin to sexism and racism. This is supported by considerable psychological evidence that most people, including doctors, are poor at predicting how they would feel if disabled, as well as by survey evidence suggesting that disabled people have high degrees of life‐satisfaction.^62^ Whether utilitarians should prioritize the abled over the disabled depends on how this issue is resolved.

Finally, *contractualism* is a theory Rawls himself supported. A simplified version of Rawls’ view, as applied to the present context, would say that the morally just course of action is the one we would choose if we did not know who we would be in the dilemma under consideration: in this case, the passengers or the pedestrians. This is choice from behind the ‘veil of ignorance’. Contractualism would support saving the greater number: we would have a greater chance of surviving since we do not know whether we would be a pedestrian or passenger. It would also reject sex as a criterion: if you did not know whether you would be a man or a woman, you would want an equal chance for each.

What about age? It is plausible that contractualists, like utilitarians, would prefer to save the younger: that version of themselves would have had less life and have more expected life to look forward to. However, data from an independent empirical study suggest that people’s preferences when behind a veil of ignorance are likely to be more complex. In that ‘Intensive Care Lifeboat’ study, participants had to choose which of two infants would be saved in situations of scarce resources.^63^ Interestingly, while participants prioritized those who will live longer over those who will die young when significant age differences were involved, they largely refrained from doing so when the differences in life expectancy were small (e.g. 40 vs. 41 years). In those cases, participants tended to prefer to decide via a coin toss.^64^ This might suggest that the degree of difference is relevant: saving a 10‐year‐old vs. an 80‐year‐old (here we might adopt the utilitarian solution and save the 10‐year‐old) is different from saving the 10‐year‐old vs. a 20‐year‐old (here we might toss a coin).

Another finding from this research was that respondents also always took disability into account—always prioritizing the abled over the disabled, even when the disability was mild.^65^ How we regard this preference will again depend on whether, and to what extent, disability is indeed a disadvantage. Those who hold that disability is a mere difference would regard such a public preference as mere prejudice and as reflecting ‘epistemic injustice’—perhaps to be screened out before we begin practical reflective equilibrium. Arguably, however, even if we hold that some disabilities are associated with disadvantage that is not exclusively due to social prejudice, a contractualist perspective may again distinguish between very significant disability (e.g. being in an irreversible state of unconsciousness) and merely mild disability (e.g. being deaf or blind). Such a preference from behind a veil of ignorance seems plausible: if you did not know if you were the blind person (or 40‐year‐old) or sighted person (or 30‐year‐old), you might prefer to toss a coin. But if you did not know if you were the permanently unconscious (or minimally conscious) person or a normally conscious person, you would strongly prefer to save the healthy version of yourself. Yet there would be no such reason to prefer one sex over another: those lives would have broadly equal predicted future value.

Which policy should we form? All three theories reject gender as a relevant factor. Utilitarianism and contractualism both converge on taking numbers into account. This also happens to be a strong public preference. The Kantian approach is here wildly inconsistent with robust global public preferences. As the philosopher John Taurek famously suggested, when faced rescuing a life boat with one person or a different one with five people, strict egalitarianism requires tossing a coin: this gives each person an equal chance of what he or she needs.^66^ But few people, and few other theories, would support such radical egalitarianism. Most people do accept that trade‐offs are sometimes necessary, even if tragic. As an illustration, in the ‘Intensive Care Lifeboat’ study mentioned above, only two out of 109 opted to toss a coin to decide whether to save one life or five; the other 107 all opted to save the greater number.^67^

Both utilitarianism and contractualism consider age and disability as potentially relevant. But these are subjects of further deliberation and empirical research. Perhaps they should be considered only to a degree: as with age, significant differences in length or quality of life ought to be taken into account, but smaller degrees should not. Robust data on the link between specific disabilities and well‐being need to be collected. But severe disabilities such as permanent unconsciousness or even profound cognitive impairment are arguably potentially relevant considerations. For example, infants with Trisomy 18 and severe intellectual disability are not offered life‐saving heart transplants in part because of limitations of resources.^68^ It is notable that the ‘Intensive Care Lifeboat’ study found no difference between utilitarian and veil of ignorance formulations of saving dilemmas on two test scenarios.^69^ This is in line with a recent psychological study that found that choices under a veil of ignorance often closely mirror utilitarian ones.^70^

As this brief discussion illustrates (as can be visulaised in Table 2), some preferences (e.g. relating to sex, or for that matter to ethnicity or religion) can be rejected regardless of their degree of public support because they cannot be justified in terms of any reasonable ethical framework. Others (such as concern for numbers) cohere with multiple if not all ethical theories; according to CREP, in such cases the preference still has a strong claim to shape policy. In other cases (such as disability), the apparent match between preferences and theories is inconclusive since it may be based on mistaken empirical views (e.g., about the relationship between disability and quality of life).

**TABLE 2:**
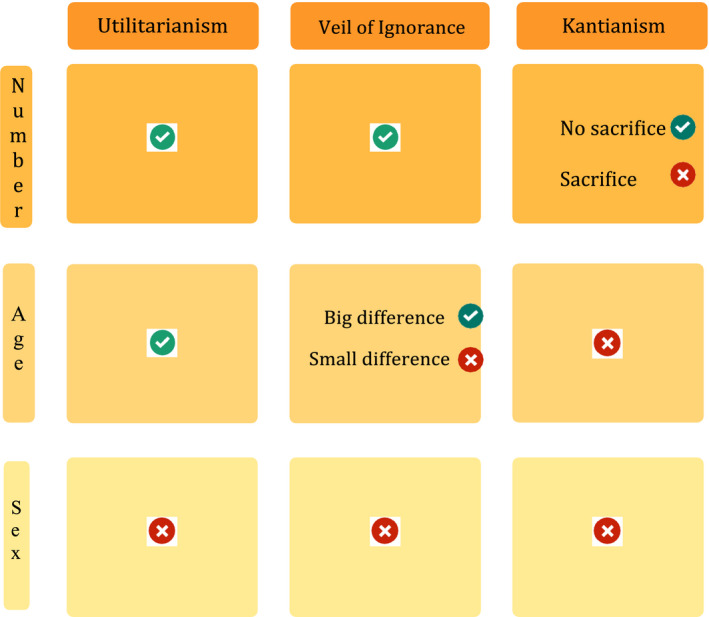
Three ethical approaches to programming autonomous vehicles

In this way, CREP provides a way to take public preferences into account in an ethically robust way. Ethical theory can also suggest potentially important factors for public deliberation: it should play a greater role in shaping the initial data‐gathering process. For example, Awad et al. did not report on whether and how responsibility for risk ought to be considered—a factor emphasized both by the German Government Report and in John Harris’s critique of the Awad et al. study.^71^ Utilitarianism and contractualism might widely diverge on how responsibility ought to be taken into account, with utilitarians only concerned with the short‐ and long‐term consequences of prioritizing the non‐responsible over the responsible, whereas contractualists can directly appeal to what different parties deserve.

The use of responsibility in the allocation of limited healthcare resources (such as organs or surgery) is a lively and controversial topic reflecting consequentialist and desert‐based approaches,^72^ which has direct relevance to allocation of harm and risk in AVs. Likewise, practical reflective equilibrium promises to advance debate on the use of public preferences around responsibility in organ allocation^73^ and healthcare generally beyond the application of novel technologies.

There is a further way in which ethical input is critical in this context. Many real‐life decisions involving artificial intelligence (AI) will be the result of an incredibly complex computational process that will be opaque to human observers, including AI experts. Once AVs are programmed to make ethically loaded choices—whether via practical reflective equilibrium or otherwise—it is crucial that the real‐life outputs of such algorithms be collected for ethical audit. The ‘decisions’ made by AVs need to make ethical sense in light of our best ethical theories. If they do not, we have reason to conclude that the algorithms that generated these decisions are morally faulty. Thus, moral philosophy is as critical to assessing what we get out of AI as it is to deciding what to put into it.

## 6 CONCLUSION

Big data, AI and machine learning afford unprecedented opportunities to obtain data about people’s explicit and implicit preferences, and to derive their values from their behaviour. How should such preferences and intuitions play a role in shaping policy and law? Technology, such as AVs and AI, creates an urgent need to revise existing laws and policies or to develop new ones.

It is important that we do not slide into the allure of using technology to avoid difficult ethical issues and questions. We should not return to the ‘tyranny of the majority’ or, in contemporary terms, the ‘tyranny of big data’. Data about preferences and behaviour are important. Ultimately, in a democracy, policymakers are accountable and responsible to the people. But nonetheless we should not fall victim to the ‘naturalistic fallacy’ of confusing facts with values. The ethical enterprise requires a distinctive kind of reasoning. Our proposed procedure of CREP attempts to capture this.

Both public preferences and ethical theories, concepts and principles are necessary for moral progress. Where there is reasonable philosophical disagreement, public preferences that have been formed after minimizing bias and prejudice have an important role to play in determining policy. But where there is robust philosophical agreement across multiple ethical perspectives, public preferences no matter how widespread should not rule the day. Preferences do not necessarily track value, and moral progress often requires reshaping preferences. CREP offers one way of combining preference/intuition and philosophical theory in relation to policy proposals. Ultimately, though, in a democracy these will be subject to the will of the people.

## Footnotes

^1^

We briefly sketched the basic idea in Savulescu, J., Kahane, G., & Gyngell, C. (2019). From public preferences to ethical policy. *Nature Human Behaviour*, *3*(12), 1241–1243. 10.1038/s41562-019-0711-6 31451736

^2^

Belvedere, M. J. (2017, Jan 9). Ford aims for self‐driving car with no gas pedal, no steering wheel in 5 years, CEO says. Retrieved from https://www.cnbc.com/2017/01/09/ford‐aims‐for‐self‐driving‐car‐with‐no‐gas‐pedal‐no‐steering‐wheel‐in‐5‐years‐ceo‐says.html [Accessed Dec 13, 2019].

^3^

Litman, T. (2015). Autonomous Vehicle Implementation Predictions: Implications for Transport Planning: Report for the Victoria Tr, Policy Inst. Available at https://www.vtpi.org/avip.pdf [Accessed Apr 9, 2021].

^4^

Bagloee, S. A., Tavana, M., Asadi, M., & Oliver, T. (2016). Autonomous vehicles: Challenges, opportunities, and future implications for transportation policies. *Journal of Modern Transportation*, *24*(4), 284–303. 10.1007/s40534-016-0117-3

^5^

Foot, P. (1967). The problem of abortion and the doctrine of double effect. *Oxford Review*, *5*, 5–15.

^6^

See e.g. Wood, A. (2011). Humanity as an end in itself. In D. Parfit (Ed.), *On what matters*. Vol. 2. Oxford, UK: Oxford University Press.

^7^

To simplify the presentation, we will write as if AVs make decisions. But in doing so we do not intend to take any stance on whether the outputs of such programs count as genuine choices.

^8^

Awad, E., Dsouza, S., Kim, R., Schulz, J., Henrich, J., Shariff, A., & Rahwan, I. (2018). The Moral Machine experiment. *Nature*, *563*(7729), 59.10.1038/s41586-018-0637-630356211

^9^

Ibid.

^10^

For an extended argument for why policy‐makers should not endorse many of the preferences uncovered by the moral machines experiment, see Harris, J. (2020). The immoral machine. *Cambridge Quarterly of Healthcare Ethics, 29*(1), 71–79. 10.1017/S096318011900080X 31581972

^11^

See e.g. Cikara, M., Farnsworth, R. A., Harris, L. T., & Fiske, S. T. (2010). On the wrong side of the trolley track: Neural correlates of relative social valuation. *Social Cognitive and Affective Neuroscience*, *5*(4), 404‐413; Swann Jr, W. B., Gómez, Á., Dovidio, J. F., Hart, S., & Jetten, J. (2010). Dying and killing for one’s group: Identity fusion moderates responses to intergroup versions of the trolley problem. *Psychological Science*, *21*(8), 1176–1183.

^12^

Singer, P. (1974). Sidgwick and reflective equilibrium. *Monist*, *58*(3), 516.

^13^

See e.g. Sinnott‐Armstrong, W. (Ed.) (2008). Framing moral intuitions. In *Moral psychology,* *Vol* *2: The cognitive science of morality: Intuition and diversity* (pp. 47–76). Cambridge, MA: MIT Press.

^14^

Pastötter, B., Gleixner, S., Neuhauser, T., & Bäuml, K.‐H. T. (2013). To push or not to push? Affective influences on moral judgment depend on decision frame. *Cognition*, *126*(3), 373–377.10.1016/j.cognition.2012.11.00323280150

^15^

Cao, F., Zhang, J., Song, L., Wang, S., Miao, D., & Peng, J. (2017). Framing effect in the trolley problem and footbridge dilemma. *Psychological Reports*, *120*(1), 88–101.10.1177/003329411668586628558527

^16^

Sleeman, K. (2017). The murky issue of whether the public supports assisted dying. Retrieved from http://theconversation.com/the‐murky‐issue‐of‐whether‐the‐public‐supports‐assisted‐dying‐85279 [Accessed Dec 13, 2019]. The authors doubt this claim.

^17^

Mill, J. S. (2002). *On liberty*. Mineola, NY: Dover Publications.

^18^

Singer (1974), op. cit.

^19^

Harris (2019), op. cit., p. 5.

^20^

Harris (2019), op. cit., p. 7.

^21^

See e.g. Thomson, J. J. (2008). Turning the trolley. *Philosophy and Public Affairs,* *36*, 359–374.

^22^

See McMahan, J. (2013). Moral intuition. In H. LaFollette & I. Persson (Eds.), *The Blackwell guide to ethical theory*, 2nd edn (pp. 103–120). Malden: Blackwell.

^23^

Schwitzgebel, E., & Cushman, F. (2012). Expertise in moral reasoning? Order effects on moral judgment in professional philosophers and non‐philosophers. *Mind and Language,* *27*(2), 135–153.

^24^

See, for example, McMahan (2013), op. cit.

^25^

Another assumption we are making is that the current empirical evidence does not show that moral intuitions are generally unreliable. In fact, we think that claims to the contrary greatly exaggerate the debunking force of recent psychological work. On this, see May, J. (2018). *Regard for reason in the moral mind*. Oxford, UK: Oxford University Press.

^26^

Sidgwick famously highlighted the significance of convergence between people and across time in *The Methods of Ethics*. For a contemporary defence of this claim, see Huemer, M. (2008). Revisionary intuitionism. *Social Philosophy and Policy,* *25*(1), 368–392. Huemer writes that we should ‘[e]schew intuitions that are not widely shared, that are specific to one’s own culture, or that entail positive evaluations of the practices of one’s own society and negative evaluations of the practices of other societies’.

^27^

Notice that claiming that wide public convergence on certain moral intuitions provides greater pro tanto support to a moral view is compatible with thinking that the moral intuitions of ethicists are typically superior; notice that even here much greater weight is usually given to those intuitions that are widely shared amongst ethicists.

^28^

This seems to be what Awad et al. have in mind when they write that ‘Any attempt to devise artificial intelligence ethics must be at least cognizant of public morality’ because ordinary people may strongly disagree, and reject, the prescriptions of ethicists. However, Awad et al. also write that in such a case the work of ethicists would be ‘useless’. This verdict is mistaken in multiple ways. As we shall see below, there can be cases where we *should* simply override or ignore public views, even if these are widespread. Notice that such considerations about political legitimacy could still support the proposal we develop below even to those who do not accept the previous point about intuitions having justificatory force.

^29^

https://reports.weforum.org/global‐risks‐2015/.

^30^

Baylis, F. (2019). *Altered inheritance: CRISPR and the ethics of human genome editing*. Cambridge, MA: Harvard University Press.

^31^

Bonnefon, J.‐F., Shariff, A., & Rahwan, I. (2016). The social dilemma of autonomous vehicles. *Science*, *352*(6293), 1573–1576.10.1126/science.aaf265427339987

^32^

Rawls, J. (1951). Outline of a decision procedure for ethics. *Philosophical Review*, *60*(2), 77–197.

^33^

Daniels, N. (1979). Wide reflective equilibrium and theory acceptance in ethics. *Journal of Philosophy, 76*, 256–282.

^34^

Rawls, J. (1971). *A theory of justice*. Oxford: Oxford University Press. p. 21.

^35^

Singer (1974), op. cit.; McPherson, T. (2015). The methodological irrelevance of reflective equilibrium. In C. Daly (Ed.), *The Palgrave handbook of philosophical methods* (pp. 652–674). Basingstoke, UK: Palgrave Macmillan.

^36^

Scanlon, T. M. (2002). Rawls on justification. In S. Freeman (Ed.) *The Cambridge companion to Rawls* (pp. 139–167). Cambridge, UK: Cambridge University Press; Arras, J. (2007). The way we reason now: Reflective equilibrium in bioethics. In B. Steinbock (Ed.), *The Oxford handbook of bioethics* (pp. 46–71). New York, NY: Oxford University Press.

^37^

This is explicit in Daniels (1979), op. cit., p. 258.

^38^

Arras, J. D., & Brody, H. (2013). Methods of practical ethics. In H. Lafollette (Ed.), *International encyclopedia of ethics* (p. wbiee768). Oxford, UK: Blackwell Publishing Ltd. 10.1002/9781444367072.wbiee768

^39^

Arras & Brody (2013), op. cit. Tersman sees this worry as the ‘most troubling’ objection to the approach. Tersman, F. (2018). Recent work on reflective equilibrium and method in ethics. *Philosophy Compass*, *13*(6), e12493. 10.1111/phc3.12493

^40^

We believe that the process we will outline meets both criteria, though we admit that they can potentially be in tension and that giving more emphasis to one of the two could lead to different policies. To avoid contentious metaethical commitments, we set aside the question of whether the process also helps us to arrive at moral truth or is rather a way for communities to work out practical dilemmas (see also Arras & Brody (2013), op. cit.).

^41^

Ibid.

^42^

It might be asked whether such screening does not already involve appeal to substantive ethical considerations of the sort that are supposed to be brought in only at the next stage of CREP, raising worries about circularity. However, while some attempts to ‘debunk’ certain moral intuitions may already assume contested ethical assumptions, this need not be the case. Framing effects, for example, reveal inconsistency in intuitions about substantively identical scenarios. In other cases, screening can legitimately appeal to moral claims so long as these are not contested. If, for example, we discover that a certain intuition is driven only by the skin colour of a protagonist, then we do not need to consult ethical theories to dismiss it. For further discussion of these larger issues, see Kahane, G. (2016). Is, ought, and the brain. In S. M. Liao (Ed.), *Moral brains: The neuroscience of morality* (pp. 281–311). Oxford: Oxford University Press.

^43^

Such screening would be pointless if our intuitions were incorrigibly unreliable. As explained earlier, we assume that existing psychological evidence is far from showing that this is the case. We concede that if such evidence were to emerge, the method we propose would at most offer political legitimacy, not moral justification. For discussion of similar questions about ‘traditional’ reflective equilibrium, see Paulo, N. (2020). The unreliable intuitions objection against reflective equilibrium. *The Journal of Ethics*, 24(3), 333–353. 10.1007/s10892-020-09322-6, and Tersman (2018), op. cit., note 39.

^44^

Bowman‐Smart, H., Savulescu, J., Mand, C., Gyngell, C., Pertile, M. D., Lewis, S., & Delatycki, M. B. (2019). ‘Is it better not to know certain things?’: Views of women who have undergone non‐invasive prenatal testing on its possible future applications. *Journal of Medical Ethics*, *45*(4), 231–238.10.1136/medethics-2018-105167PMC658274230679192

^45^

Mansfield, C., Hopfer, S., & Marteau, T. M. (1999). Termination rates after prenatal diagnosis of Down syndrome, spina bifida, anencephaly, and Turner and Klinefelter syndromes: A systematic literature review. European Concerted Action: DADA (Decision‐making After the Diagnosis of a fetal Abnormality). *Prenatal Diagnosis*, *19*(9), 808–812.10521836

^46^

See, e.g., Bostyn, D. H., Sevenhant, S., & Roets, A. (2018). Of mice, men, and trolleys: Hypothetical judgment versus real‐life behavior in trolley‐style moral dilemmas. *Psychological Science*, *29*, 1084–1093; Francis, K.B., Terbeck, S., Briazu, R.A. *et al*. (2017). Simulating moral actions: An investigation of personal force in virtual moral dilemmas. *Sci* *Rep*, *7*, 13954.10.1177/095679761775264029741993

^47^

For an attempt to use virtual reality to directly study ethical decisions about autonomous vehicles, see Sütfeld, L. R., Gast, R., König, P., & Pipa, G. (2017). Using virtual reality to assess ethical decisions in road traffic scenarios: Applicability of value‐of‐life‐based models and 85 influences of time pressure. *Front*. *Behav. Neurosci*., *11*, 122.10.3389/fnbeh.2017.00122PMC549695828725188

^48^

Here we agree with Harris (2020), op. cit., note 10, who writes that a solution to a moral dilemma ‘has to show how the circumstances which make it a moral dilemma, have been weighed carefully one against another, and morally persuasive reasons, facts and/or justifications found for having a moral preference for one outcome rather than another’. Importantly, however, this does not entail that public preferences have no role to play in the process—especially when its output is meant to be a practicable policy as opposed to a personal view about the morally correct solution.

^49^

See Bykvist, K. (2017). Moral uncertainty. *Philosophy Compass*, *12*(3), e12408.

^50^

For example, see Dryzek, J. S., & Niemeyer, S. (2010). *Deliberative turns. Foundations and frontiers of deliberative governance*. Oxford, UK: Oxford University Press. Retrieved from http://www.oxfordscholarship.com/view/10.1093/acprof:oso/9780199562947.001.0001/acprof‐9780199562947‐chapter‐1

^51^

Elstub, S. (2018). Deliberative and participatory democracy. In S. Elstub, A. Bächtiger, J.S. Dryzek, J. Mansbridge & M. Warren (Eds.). *The Oxford Handbook of deliberative democracy* (pp. 186–202). Oxford, UK: Oxford University Press.

^52^

Carson, L., & Elstub, S. (2019). Comparing participatory and deliberative democracy. *New Democracy* *Research and Development note,* Available at https://www.newdemocracy.com.au/wp‐content/uploads/2019/04/RD‐Note‐Comparing‐Participatory‐and‐Deliberative‐Democracy.pdf [Accessed 27 July 2020].

^53^

Wilsdon, J., & Willis, R. (2004). *See‐through science: Why public engagement needs to move upstream*. London, UK: Demos.

^54^

Solomon, S., & Abelson, J. (2012). Why and when should we use public deliberation? *Hastings Center Report*, *42*(2), 17–20.10.1002/hast.27PMC364548322733325

^55^

Garard, J., Koch, L., & Kowarsch, M. (2018). Elements of success in multi‐stakeholder deliberation platforms. *Palgrave Communications*, *4*(1), 1–16.

^56^

Abelson, J., Forest, P.‐G., Eyles, J., Smith, P., Martin, E., & Gauvin, F.‐P. (2003). Deliberations about deliberative methods: Issues in the design and evaluation of public participation processes. *Social Science & Medicine*, *57*(2), 239–251.10.1016/s0277-9536(02)00343-x12765705

^57^

Cf. Harris, J. (2001). *The value of life: An introduction to medical ethics* (Repr). London, UK: Routledge.

^58^

Arora, C., Savulescu, J., Maslen, H., Selgelid, M., & Wilkinson, D. (2016). The intensive care lifeboat: A survey of lay attitudes to rationing dilemmas in neonatal intensive care. *BMC Medical Ethics*, *17*(1), 69. 10.1186/s12910-016-0152-y. While this was an online study of a far smaller scale than that of Awad et al., it seems likely that a similar pattern of response would have been found on the global scale.PMC510021127821118

^59^

Ethics Commission. (2017). *Automated and connected driving*. Retrieved from https://www.bmvi.de/SharedDocs/EN/publications/report‐ethics‐commission.pdf. While the German commission’s report is clearly driven by Kantian concerns, we do not claim that this is the only way to apply Kantian ideas to AVs.

^60^

Ibid.

^61^

See Harris, J. (1987). QALYfying the value of life. *Journal of Medical Ethics*, *13*(3), 117–123, for a critique.10.1136/jme.13.3.117PMC13756583669036

^62^

See, for example, Bach, J. R., & Tilton, M. C. (1994). Life satisfaction and well‐being measures in ventilator assisted individuals with traumatic tetraplegia. *Archives of Physical Medicine and Rehabilitation*, *75*(6), 626–632; Saigal, S., Feeny, D., Rosenbaum, P., Furlong, W., Burrows, E., & Stoskopf, B. (1996). Self‐perceived health status and health‐related quality of life of extremely low‐birth‐weight infants at adolescence. *JAMA*, *276*(6), 453–459; Albrecht, G. L., & Devlieger, P. J. (1999). The disability paradox: High quality of life against all odds. *Social Science & Medicine*, *48*(8), 977–988.

^63^

See Arora et al., op. cit note 9.

^64^

Ibid: note 33.

^65^

Ibid: note 33.

^66^

Taurek, J. M. (1977). Should the numbers count? *Philosophy and Public Affairs*, *6*(4), 293–316.11662404

^67^

Ibid: note 33. Additional File Survey Monkey Dataset

^68^

Savulescu, J. (2001). Resources, Down’s syndrome, and cardiac surgery. *BMJ*, *322*(7291), 875–876.10.1136/bmj.322.7291.875PMC112005411302884

^69^

Ibid: note 33.

^70^

Huang, K., Greene, J. D., & Bazerman, M. (2019). Veil‐of‐ignorance reasoning favors the greater good. *Proceedings of the National Academy of Sciences*, *116*(48), 23989–23995.10.1073/pnas.1910125116PMC688382431719198

^71^

Harris writes as if prioritization choices that AVs would need to take always reflect a situation where the AV is out of control and must choose whether to sacrifice the occupants or innocent bystanders. While this may fit the way Awad et al. present their scenarios, there will also be cases where the AV is ‘blameless’, so to speak, or when it must choose whether to collide into one of several groups of bystanders. Moreover, it is not at all clear that the occupants of an AV should bear the costs of the AV losing control—in most such cases, they will be as blameless as the bystanders outside the car. Finally, we should bear in mind that if governments require that AVs always sacrifice their occupants then this will almost certainly massively reduce the use of AVs, leading to a much higher chance of death by vehicle for everyone.

^72^

Pillutla, V., Maslen, H., & Savulescu, J. (2018). Rationing elective surgery for smokers and obese patients: responsibility or prognosis? *BMC Medical Ethics*, *19*(1), 28; Savulescu, J. (2018). Golden opportunity, reasonable risk and personal responsibility for health. *Journal of Medical Ethics*, *44*(1), 59–61; Brown, R. C. H., & Savulescu, J. (2019). Responsibility in healthcare across time and agents. *Journal of Medical Ethics*, *45*(10), 636–644.10.1186/s12910-018-0272-7PMC592197329699552

^73^

Freedman, R., Borg, J. S., Sinnott‐Armstrong, W., Dickerson, J. P., & Conitzer, V. (2020). Adapting a kidney exchange algorithm to align with human values. *Artificial Intelligence*, *283*, 103261. 10.1016/j.artint.2020.103261

